# Supplementary material for: Comparison of indoor temperatures of homes with recommended temperatures and effects of disability and age: an observational, cross-sectional study
Source: BMJ Open. 2018 May 14;8(5):e021085. doi: 10.1136/bmjopen-2017-021085 (PMC5961609; doi:10.1136/bmjopen-2017-021085)
Supplement: Author's manuscript [file bmjopen-2017-021085.draft_revisions.pdf]

BMJ Open is committed to open peer review. As part of this commitment we make the peer review history of every article we publish publicly available.

When an article is published we post the peer reviewers' comments and the authors' responses online. We also post the versions of the paper that were used during peer review. These are the versions that the peer review comments apply to.

The versions of the paper that follow are the versions that were submitted during the peer review process. They are not the versions of record or the final published versions. They should not be cited or distributed as the published version of this manuscript.

BMJ Open is an open access journal and the full, final, typeset and author-corrected version of record of the manuscript is available on our site with no access controls, subscription charges or pay-per-view fees (http://bmjopen.bmj.com).

If you have any questions on BMJ Open's open peer review process please email info.bmjopen@bmj.com

BMJ Open

A comparison of indoor temperatures of homes to recommended temperatures and effects of disability and age – an observational, cross-sectional study

Journal:	*BMJ Open*
Manuscript ID	bmjopen-2017-021085
Article Type:	Research
Date Submitted by the Author:	11-Dec-2017
Complete List of Authors:	Huebner, Gesche; UCL, Energy Institute<br>Hamilton, Ian; University College London, UCL Energy Institute<br>Chalabi, Zaid; London School of Hygiene and Tropical Medicine, Social and Environmental Health Research<br>Shipworth, David; Energy Institute<br>Oreszczyn, Tadj; University College London, UCL Energy Institute
Keywords:	EPIDEMIOLOGY, PUBLIC HEALTH, Adverse events < THERAPEUTICS

SCHOLARONE™
Manuscripts

Title:

A comparison of indoor temperatures of homes to recommended temperatures and effects of disability and age – an observational, cross-sectional study

Authors:

G. M. Huebner (corresponding author)
Senior Research Associate
g.huebner@ucl.ac.uk
Energy Institute
University College London
London, WC1E 6BT
UK

Ian Hamilton
Reader
Energy Institute
University College London
London, WC1E 6BT
UK

Zaid Chalabi
Associate Professor
Department of Social and Environmental Health Research
London School of Hygiene and Tropical Medicine
London
WC1H 9SH
UK

David Shipworth
Professor
Energy Institute
University College London

For peer review only

London, WC1E 6BT
UK

Tadj Oreszczyn
Professor
Energy Institute
University College London
London, WC1E 6BT
UK

For peer review only

Abstract

Objectives
This study examines if measured temperatures in winter in English meet the recommendation of having at least 18°C at all times and where occupants are either aged above 64 years or have a long-term disability.

Design.
Cross-sectional, observational study.

Setting
England.

Participants.
635 households.

Outcomes measures.
(1) Mean temperature for each room, (2) Proportion of days for each room meeting the criterion, (3) Average hours in each room at 18°C, (4) Average hours at night at 18°.

Results
Mean temperatures in the bedroom were M_{BR} = 18.15°C (SD = 2.51), the living room M_{LR} = 18.90°C (SD = 2.46), and the hallway M_{Hall}= 18.25°C (SD = 2.57).

The median number of days meeting the criterion was between 19 and 31%. For the living room, a higher share of days meet the criterion in the group with a LTD ($M_{disability}$ = 342 vs. $M_{no\_disability}$ = 301; 95% CI: 8 - 74) and when someone over 64 years was present ($M_{above64}$ = 341, $M_{below65}$ = 301; 95% CI: 8 - 74).

The median number of hours per day meeting the criterion was between 13 and 17. In the living room, households with a disability had more hours at 18°C ($M_{disability}$ = 364, $M_{no\_disability}$ = 297, 95% CI: 17 – 83) as did those within the older age group ($M_{above64}$ = 347, $M_{below65}$ = 296, 95% CI: 18 – 84). In the hallway, more hours meet the criterion in those with a LTD ($M_{disability}$ = 338, $M_{no\_disability}$ = 302, 95% CI: 3 – 70).

247 homes had at least nine hours of at least 18°C at night; no effect of age or disability.

For peer review only

Conclusions.

Many households are at risk of negative health outcomes. Energy use would increase substantially if keeping homes at 18°C, without significant improvement in their energy performance.

Strengths and limitations of the study

- This is the first analysis that allows examining the specific objective of comparing empirical temperature measurements to recommendations.
- The data set used corresponds to a largely representative sample in England.
- Despite outlier correction, it is possible that days were retained in the data set in which the dwelling was empty.
- Only three rooms in the homes were monitored as opposed to every room in a house.
- All three winter months were relatively mild; it is likely that colder winters would mean reveal an even greater discrepancy between recommendations and realized temperatures.

For peer review only

Introduction

The 2016 Cold Weather Plan for England recommended 18°C as day- and night temperature for those 65 and older or anyone with pre-existing medical conditions (1); and a recent systematic review on the link between internal temperatures in homes and health concluded that results from the retrieved studies were sufficient to recommend a temperature of at least 18°C for the whole population at all times (2). The 18°C threshold was judged particularly important for people over 65 years or with pre-existing medical conditions, with a particular emphasis on it being kept at night.

The need for an indoor temperature threshold arises from the burden of excess winter mortality in England; 15% more deaths occur in winter months than non-winter months, corresponding to about 24,000 extra deaths per winter (3), significantly higher than in other European countries (4). Whilst a net of complex factors impacts on winter deaths, the poor state of housing and health inequalities are major reasons for the higher excess winter death rate in England (2,5). Excess winter deaths increase significantly with age of occupants, age of the property, and poorer thermal efficiency ratings, and are associated with lower indoor temperatures (5,6). This paper examines to what extent homes in England meet temperature recommendations in winter by comparing empirical data from 635 homes to the blanket recommendation of 18°C as suggested (2).

Temperatures vary widely between homes and over the course of a day (7). Average temperatures during the heating season were 19.3°C for the living room, 18.8°C for the hallway and 18.9°C for the bedroom (8). Whilst these indoor temperatures are above the recommended 18°C, they reflect the average across homes and days. Given the known variability between homes, a substantial number of homes likely had temperatures below the recommendation. Analysis of indoor temperature during cold conditions have shown considerable variability in temperatures among older households that is modified by dwelling energy performance and socio-economic conditions (9).

To our knowledge, no study has assessed to what extent homes in England meet the recommended temperatures. The objectives of this paper are to investigate:

- Number of days in winter meeting the criterion.
- Average number of hours per day meeting the criterion.
- Average number of hours per night meeting the criterion.

For peer review only

- Comparison of the above metrics depending on whether someone in the household has a long-term disability or is over 64 years.

This paper does not aim at explaining reasons behind the differences found, e.g. whether they are due to housing factors, income, personal choice, etc. but focuses on assessing the "status quo" i.e. situation as measured in the study.

Methods

Data.

This study used data from the 2011 Energy Follow Survey (EFUS) commissioned by the Department of Business, Energy and Industrial Strategy (BEIS) (then the Department of Energy and Climate Change) (10), a large-scale cross-sectional national survey in England, and its parent-survey, the English Housing Survey (EHS), a national survey of people's housing circumstances, characteristics and condition. The EFUS survey consisted of an interview survey of a sub-set of households (N =2,616) that had been first visited as part of the 2010/2011 EHS. A sub-set of those interviewed (N = 943) consented to having temperature loggers in up to three rooms of the house set to record temperatures every 20 minutes from February 2011 to January 2012. Whilst it is impossible to know if there was any systematic difference in temperature between those who consented to loggers and those who did not, this is unlikely given that the households with loggers were broadly representative in regards to Census data (see Table 1).

The linked data sets were explicitly made available by BEIS for this research project. Parts of the dataset used in this study remain private (i.e. the high-resolution temperature data and the connection identifier between the EFUS and EHS). The non-linked data sets and summarised temperature data are available on UK Data Archives. As this paper constitutes secondary data analysis, no ethical approval was required and no personal data (i.e. identifying individuals) was available or used.

Valid temperature data was obtained from N = 823 households (see (10) for details). For this paper, only those N = 760 households were considered with temperature measurements in all three rooms (bedroom, living room, hallway) that were to be monitored. 105 households were excluded because of changes to the household or home since the last EHS. Hence, the final sample size on which all analyses are based is N = 635 homes with approximately national representativeness on geographical location, tenure and dwelling type (see Table 1).

For peer review only

Table 1. Comparison of sample characteristics to 2011 Census data.

	N in sample	% in sample	% in 2011 census
**Region** (11)			
North East	44	6.93	4.90
North West	103	16.22	13.30
Yorkshire & Humber	83	13.07	9.97
East Midlands	53	8.35	8.55
West Midlands	58	9.13	10.57
East	88	13.86	11.03
London	46	7.24	15.42
South East	101	15.91	16.29
South West	59	9.29	9.98
**Dwelling type** (12)			
Detached	153	24.09	22.30
Semi-detached	204	32.13	30.70
Terraced (including end-terrace)	178	28.03	24.50
Purpose-built flats	86	13.54	16.70
Converted flat	14	2.20	4.30
In commercial building	0	0.00	1.10
Caravan, mobile home etc.	0	0.00	0.40
**Tenure** (13)			
Owned outright	192	30.24	30.60
Owned with a mortgage / loan	211	33.23	32.80
Shared ownership	na	na	0.80
Rented from council (Local Authority)	82	12.91	9.40
Social rented: Other	94	14.80	8.30
Private rented	56	8.82	16.80
Living rent free	na	na	1.30

For peer review only

Survey interview data for EHS and EFUS.

Data were collected through computer-assisted personal interviewing in the home of the respondent. For the purpose of this study, only questions relating to age of the householder and their self-reported health were analysed.

Respondents were asked if they and other household members, where applicable, had any long-standing physical or mental health condition. If the question condition was affirmed, the interviewer asked for a specification (Table 2).

Table 2. Prevalence of long-term disabilities in the sample.

Long-term disability type	Number of households in which prevalent
Vision	28
Hearing	24
Learning	12
Heart	75
Breathing	92
Mobility	146
Mental	35
Other	165
Don't know	4

Note that in some households multiple LTD existed, i.e. the 581 occurrences listed here were distributed across 369 households.

For peer review only

Of the N = 635 households, N = 369 reported one or more long-term disability (LTD). For the purpose of this study, only the dichotomized variable of "any long-term disability" vs "no long-term disability" was used, irrespective of the type of condition and total number of individuals with LTDs in one household. Any LTD indicates vulnerability in the household and an adaptation of the environment would be required.

The second variable of interest was age of the oldest household member; age was dichotomized into "64 years and younger" and "65 years and older", because 65 years was

the cut-off used for the specific recommendations on indoor temperatures (1). Among the N = 635 households, N = 206 dwellings had the oldest household member of age 65 or older.

Temperature recordings.

Temperatures were recorded using modified TinyTag Transit 2 data loggers, that have an accuracy of +/-0.2°C, a resolution of 0.01°C, and can log from -70°C to + 40°C. The temperature loggers were usually installed by the interviewer at the end of the EFUS interview, on an internal wall, away from heat sources and direct sunlight, at a height accessible by the householder but out of reach of small children (10).

Temperature recordings for February 2011, December 2011, and January 2012 were used, i.e. those months considered as winter by the Office for National Statistics (14) for which temperature data were available (15). For every dwelling, an extreme value correction was performed on the combined temperature data from the three months where any data point more than 1.5 interquartile ranges (IQRs) below the first quartile was removed as extremely low temperatures might reflect absence from the home. The median numbers of extreme values removed were 13, 2, and 8, for bedroom, living room, and hallway, respectively.

Derived variables.

Four outcome variables were constructed from the recorded temperature readings.

a) Mean temperature for each room over the winter period.

For each dwelling and room, the average temperature across the three winter months was calculated.

b) Days with temperatures above 18°C

We calculated the number of days in which homes met the criterion of being at least 18°C continuously. Whilst a strict interpretation of the recommendation would mean that 100% of all measurements need to be at 18°C or above (i.e. all 72 measurements), we relaxed the assumption to 94.4% of all measurements (i.e. 68 out of 72 measurement points). This is meant to take into account that brief drops in temperature are entirely plausible, e.g. due to window or door opening.

For each home, on each day, and in each room, we checked at each measurement point if the temperature was at least 18°C, with a 1 recorded if it was and a 0 if it was not. The values for each day were summed up and divided by the total number of measurements per day. If 68

For peer review only

measurements were at 18°C or above, then the resulting value would be 68/72 = 0.94, i.e. 94.4%. We calculated the percentage of days for which the temperature measurements during the day had 94.4% of values at 18°C or above.

The percentage of days meeting the criterion is reported instead of the absolute number as some homes did not have temperature data recordings for all 90 days (median was 86 days).

c) Hours at or above 18°C

For each home, on each day, and in each room, we calculated the average number of hours for which the temperature was least at 18°C per a 24-hour period. We checked if consecutive measurement, i.e. 20-minute segments, were both at least 18°C, where each day lasted from midnight to midnight the next day. This meant that two days (30th of January, 2012, 28th of February, 2011) were excluded from analysis as there was no subsequent day. For each home, we averaged the estimated daily temperature metrics across all days, separately for each room.

d) Hours at or above 18°C during night

We defined night-time as lasting from 8 pm to 8 am next day to take into account that people sleep at different times, and identified whether 20-minute segments of temperature readings (i.e. two consecutive measurement) were at 18°C or above within the 12-hour time window. As above, two days were excluded. For each home, we averaged the estimated nightly temperature metrics across all days. Only the bedroom was considered.

Statistical analysis.

For the normally distributed variable ‘mean temperature’ (outcome variable a)), a repeated measures ANOVA was used to test for differences between rooms, and a generalized linear model (GLM) with the fixed factors age and disability status and their interaction to test if temperatures differed depending on those variables. Post-hoc comparisons were Bonferroni adjusted.

The non-normally distributed outcome variables b) –d) were analysed using ANOVA on ranks (16) whereby data are transformed into ranks (averaged in the case of ties) over the entire data set, and then a parametric ANOVA is applied to the ranks. The rank 1 was assigned to the lowest value, i.e. to zero days meeting the criterion; a higher mean rank value indicates more days meeting the criterion. The main effects of age and disability were tested

For peer review only

and their interaction. The presence of an interaction effect is to be interpreted with greatest caution as the procedure is associated with an increase in Type 1-error (i.e., claiming statistical significance where there is none, see e.g. (17)) ; however, if no interaction effect is found, it can be assumed that indeed, there isn't one.

Additionally, for days at or above 18°C (outcome variable b)), relative risk was calculated following (18) for the rooms where disability or age had a significant effect to be able to easily articulate how much more likely those more vulnerable were to live at the criterion.

Results

Mean temperature for each room over the winter period.

Across all dwellings, mean temperatures in the bedroom were M_{BR} = 18.15°C (SD = 2.51), the living room M_{LR} = 18.90°C (SD = 2.46), and the hallway M_{Hall}= 18.25°C (SD = 2.57). A repeated measures ANOVA showed a main effect of room type, $F(2, 1268) = 58.41$, $p < .001$. Post-hoc comparisons showed the living room was significantly warmer than the bedroom (p < .001; mean difference: .75, 95% CI for difference: .94 – .57) and hallway (p < .001; mean difference: .65; 95% CI for difference: .57 –. 94) which did not differ significantly from each other.

Figure 1 shows the probability density function of the mean temperatures for the three rooms, created using the R package 'sm' (19). The area underneath each curve is unity.

<< insert Figure 1 about here >>

Figure 1 indicates a wide spread in mean temperatures. Whilst the average temperature (across days and homes) in all three rooms is slightly above 18°C, in a substantial number of homes it was below 18 °C. In the case of the bedroom, 286 dwellings (45%) had an average temperature below 18 °C, in the living room 209 (33%), and in the hallway 278 dwellings (44%).

In the bedroom, only the effect of disability was significant [$F(1, 631) = 4.38$, $p = .037$] with higher temperatures in the group with disability ($M_{disability}$ = 18.35; $M_{no\_disability}$ = 17.87; 95% CI for difference: .03 – .94). For the hallway, again only the effect of disability was significant [$F(1, 631) = 7.64$, $p = .006$] with higher temperatures in the disability group ($M_{disability}$ = 18.58, $M_{no\_disability}$ = 17.93). There was a strong trend for higher temperatures in the homes of the older age group (p = .059). In the living room, both the main effects of age

For peer review only

[F(1, 631 = 12.39, p < .001] and disability [F(1, 631) = 15.53, p <.001] were statistically significant. Temperature were higher in the group with disability ($M_{disability}$ = 19.37; $M_{no\_disability}$ = 18.50; 95% CI for difference: .44 – 1.30) and in the older age group ($M_{above64}$ = 19.32; $M_{below65}$ = 18.55; 95% CI for difference: .34 – 1.21).

Days with temperatures above 18°C

We analysed the number of days on which dwellings met the indoor temperature criterion. Figure 2 shows the probability density function of the distribution for the three rooms.

<< insert Figure 2 about here >>

Figure 2 indicates that the largest share of homes do not meet the criterion but that a substantial number of homes meet it on 90-100% of days. For the bedroom, 11% of homes meet the criterion on all days, and 17% on more than 90% of days. For the living room, the numbers are 15% and 24%, respectively, and for the hallway 12% and 17%. The median number of days that indoor temperatures meet the criterion on all days is Md_{BR} = 22.58% of days, MD_{LR} = 31.11%, and Md_{Hall} = 18.89%.

The ANOVA for ranks in the bedroom showed neither a main effect of age or disability nor an interaction effect. For the living room both the main effects of disability [F(1, 631) = 6.00, p = .015] and age [F(1, 631) = 6.06, p = .0114] were significant, with a higher share of days meeting the criterion in the group with a LTD ($M_{disability}$ = 342 vs. $M_{no\_disability}$ = 301; 95% CI for difference: 8 - 74) and with someone over 64 years present ($M_{above64}$ = 341, $M_{below65}$ = 301; 95% CI for difference: 8 - 74). Of those households with LTD, 26.8% had a continuous temperature above 18°C on 90% of days or more compared to 20.7% for those without LTD. Expressed as a relative risk (18), people with LTD are 1.30 times more likely to be living in dwellings where the temperature is consistently over 18°C compared to those without LTD, and people who are 65 years and above are 1.56 more likely than those below 65 years.

For the hallway, there were no significant effects. However, there was suggestive evidence of a trend towards more days meeting the criterion in the group with a long-term disability (p =.064).

For peer review only

Number of hours at which temperatures are at or above 18°C

The number of hours at or above 18°C were non-normally distributed with peaks at either extreme of zero and 24 hours. The median number of hours at / above 18°C was Md_{BR} = 14:01 hours, Md_{LR} = 16:57 hours, and Md_{Hall} = 13:24 hours.

Table 3 shows the median number of hours of temperature at the criterion for the three rooms separated by disability and age group.

Table 3. Median number of hours of temperature at the criterion for the three rooms separated by disability and age group.

	Disability status		Age	
	No LT disability	LT disability	Below 65 years	Above 64 years
Bedroom	13:10 hrs	14:05 hrs	12:56 hrs	15:09 hrs
Living room	15:13 hrs	17:59 hrs	15:37 hrs	20:01 hrs
Hallway	10:58 hrs	14:52 hrs	12:35 hrs	16:01 hrs

In the bedroom, there were no significant main or interaction effects. In the living room, both the main effect of disability [$F(1, 631) = 8.89$, $p = .003$] and of age [$F(1, 631) = 9.28$, $p = .002$] were significant, with more hours at or above 18°C in those households occupied by individuals with a disability ($M_{disability} = 364$, $M_{no\_disability} = 297$, 95% CI for difference: 17 – 83) and in the older age group ($M_{above\ 64} = 347$, $M_{below65} = 296$, 95% CI for difference: 18 – 84). In the hallway, the main effect of disability was significant [$F(1, 631) = 4.53$, $p = .034$] and the effect of age approached significance ($p = .073$) with again more hours meeting the criterion in the group with a LTD ($M_{disability} = 338$, $M_{no\_disability} = 302$, 95% CI for difference: 3 – 70) and the older group.

Night temperatures

We subsequently tested if the recommendation of nine hours of 18°C at night time was met (see Figure 3).

For peer review only - http://bmjopen.bmj.com/site/about/guidelines.xhtml

<< insert Figure 3 about here >>

Across the full sample, 247 homes (38.9%) had at least nine hours of temperatures of at least 18°C at night and 101 (15.9%) homes had less than one hour at the criterion. The median was 7:08 hrs.

An ANOVA on ranks showed neither a main effect of disability nor of age, and no interaction.

Discussion

This study is the first to establish whether measured temperature data in homes corresponds to the recommended temperatures, in the general population and the subgroups of those with a LTD and / or aged 65. Whilst average temperatures across homes in this sample is slightly above 18°C, the wide variability means that many homes have lower temperatures. Depending on room type, the recommended indoor temperature of 18°C was only met on 19 – 31% of days during the three studied winter months. Only 5% to 9% of homes met the criterion of being at least 18°C throughout the day, with up to 22% of homes meeting the criterion if set as having the recommended temperature throughout the day for at least 90% of days. Those with a disability and old age were 1.3 and 1.56 more likely to meet the condition than those without disability and the younger age group. The median number of hours per day at or above 18°C was 17 for the living room, and 14 and 13 ½, respectively, for bedroom and hallway. The median number of hours meeting the criterion were one to three hours higher in households with a disability / aged 65 and older. At night, 37% of homes had temperatures of at least nine hours at 18°C or more, with the median being 7 hrs, with no effect of age group or disability.

In summary, the majority of measures employed showed that the recommendation was not met, neither in the overall sample, nor within the subsamples of those more vulnerable to effects of cold.

Limitations and strengths of this study

Despite outlier correction, it is possible that days were retained in the data set in which the dwelling was empty, leading to an underestimation of the criterion being met assuming that it only holds for occupied times. Only three rooms in the homes were monitored as opposed to every room in a house. The study is cross-sectional, and cannot add evidence on whether low

For peer review only

temperatures are associated with poor health outcomes. All three winter months were relatively mild (mean temperatures in February 2011 1.7 °C above the 1981-2010 average; in December 2011 1.0 °C above the 1981-2010 average; January 2012 1.0 °C above the 1981-2010 average (20)); it is likely that colder winters would mean even lower prevalence of 18°C. Households consented to having temperature loggers installed; it is possible that temperatures in those households were either higher or lower than in those not giving consent.

This paper is the first analysis that allows examining the specific objective of comparing empirical temperature measurements to recommendations, showing a significant discrepancy and the need for action. The data set used corresponds to a largely representative sample in England; hence, results likely are generalizable to the whole of England.

Conclusions

In summary, data showed that the majority of homes do not meet the recommendation, neither across the whole sample or within the vulnerable subgroups. If living in homes below the temperature threshold is a determinant of cold-related ill health then many English households are at risk of developing negative health outcomes. Hence, substantial action is needed to increase temperatures in homes, be it through improvements in building fabric, extended use of heating systems, or increased thermostat set points.

From an energy demand perspective, energy use in buildings would increase substantially when keeping all homes at 18°C continuously. Without improvement in the energy performance of buildings, e.g. through fabric insulation and greater efficiency of heating systems, this outcome would result in an increase in heating energy use and move away from the UK's energy efficiency goals.

There is also the question of whether individuals can afford to increase fuel expenditure to achieve the stated indoor temperature threshold. Mean energy expenditure was 4.4% of total household expenditure, with a substantially higher proportion of 9.7% in the lowest income decile (21). Spending on fuel to increase temperatures would result in a greater proportion of household resources allocated to fuels. This increase in spending could result in a net cost-benefit if the health impacts were accounted for in these calculations (22)– but householders might not realize this directly.

For peer review only

Contributor ship statement

GMH and IH conceived the study.

GMH conducted the analysis.

ZC advised on the analysis.

GMH wrote the paper.

IH, ZC, TO, and DS proofread the manuscript multiple times.

Competing interests

The authors declare no competing interests.

Funding

Funding for this research was provided by the Research Councils UK (RCUK) Centre for Energy Epidemiology, grant reference EP/K011839/1.The funders were not involved in any aspects of the research.

Data sharing agreement

The linked data sets were explicitly made available by BEIS for this research project. Parts of the dataset used in this study remain private (i.e. the high resolution temperature data and the connection identifier between the EFUS and EHS to link dwelling and household features). The individual, non-linked data sets and the summarised temperature data are available on UK Data Archives.

For peer review only

References

1. Public Health England. The Cold Weather Plan for England Protecting health and reducing harm from cold weather About Public Health England. 2015;(October). Available from: https://assets.contentful.com/ii3xdrqc6nfw/6DFf1khXWM0agYA2ScM6EY/b5220d4552c43d70b9f1dd82194a6b84/Cold_weather_plan_2016.pdf

2. Jevons R, Carmichael C, Crossley A, Bone A. Minimum indoor temperature threshold recommendations for English homes in winter - A systematic review. Public Health [Internet]. 2016;136:4–12. Available from: http://dx.doi.org/10.1016/j.puhe.2016.02.007

3. Office for National Statistics. Excess Winter Mortality in England. 2014;14:22. Available from: https://www.ons.gov.uk/peoplepopulationandcommunity/birthsdeathsandmarriages/deaths/bulletins/excesswintermortalityinenglandandwales/2015to2016provisionaland2014to2015final#things-you-need-to-know

4. Fowler T, Southgate RJ, Waite T, Harrell R, Kovats S, Bone A, et al. Excess winter deaths in Europe: A multi-country descriptive analysis. Eur J Public Health. 2015;25(2):339–45.

5. Wilkinson P, Landon M, Armstrong B, Stevenson S, Pattenden S, Mckee M, et al. Cold comfort: The social and environmental determinants of excess winter deaths in England , 1986-96. 2001;34. Available from: http://www.jrf.org.uk/publications/cold-comfort-social-and-environmental-determinants-excess-winter-deaths-england-1986-19

6. NICE. Excess cess winter deaths and illness and the health risks associated with cold homes [Internet]. 2015. Available from: https://www.nice.org.uk/guidance/ng6/resources/excess-winter-deaths-and-morbidity-and-the-health-risks-associated-with-cold-homes-51043484869

7. Huebner GM, McMichael M, Shipworth D, Shipworth M, Durand-Daubin M, Summerfield A. The reality of English living rooms - A comparison of internal temperatures against common model assumptions. Energy Build. 2013;66:688–96.

For peer review only

8. Hulme J, Beaumont A, Summers C. Energy Follow-Up Survey 2011, Report 1: Summary of findings. BRE behalf DECC [Internet]. 2013;(289605). Available from: https://www.gov.uk/government/uploads/system/uploads/attachment_data/file/274769/1_Summary_Report.pdf

9. Hamilton IG, O'Sullivan A, Huebner G, Oreszczyn T, Shipworth D, Summerfield A, et al. Old and cold? Findings on the determinants of indoor temperatures in English dwellings during cold conditions. Energy Build [Internet]. 2017 Apr [cited 2017 Aug 22];141:142–57. Available from: http://linkinghub.elsevier.com/retrieve/pii/S0378778816310489

10. BRE. Energy Follow-Up Survey 2011, Report 11 : Methodology. 2013;(288851):1–120.

11. ONS. KS101EW Usual resident population, local authorities in England and Wales [Internet]. [cited 2017 Nov 23]. Available from: https://www.ons.gov.uk/peoplepopulationandcommunity/populationandmigration/populationestimates/datasets/2011censuskeystatisticsforlocalauthoritiesinenglandandwales

12. ONS. KS401EW Dwellings, household spaces and accommodation type, local authorities in England and Wales (Excel sheet 305Kb) [Internet]. [cited 2017 Nov 23]. Available from: https://www.ons.gov.uk/peoplepopulationandcommunity/populationandmigration/populationestimates/datasets/2011censuskeystatisticsforlocalauthoritiesinenglandandwales

13. ONS. KS402EW Tenure, local authorities in England and Wales (Excel sheet 270Kb) [Internet]. [cited 2017 Nov 23]. Available from: https://www.ons.gov.uk/peoplepopulationandcommunity/populationandmigration/populationestimates/datasets/2011censuskeystatisticsforlocalauthoritiesinenglandandwales

14. MetOffice. Winter 2011/12 [Internet]. [cited 2017 Nov 23]. Available from: https://www.metoffice.gov.uk/climate/uk/summaries/2012/winter

15. BRE. Report 2 : Mean household temperatures [Internet]. 2013. Available from: http://doc.ukdataservice.ac.uk/doc/7471/mrdoc/pdf/7471_2_mean_household_temperatures.pdf

16. Conover WJ, Iman RL. Rank Transformations as a Bridge between Parametric and

For peer review only

Nonparametric Statistics Rank Transformations as a Bridge Between Parametric and Nonparametric Statistics. Am Stat. 1981;

17. Higgins JJ, Tashtoush S. An aligned rank transform test for interaction. Nonlinear World. 1994;

18. Altmann DG. Practical Statistics for Medical Research. Chapman & Hall; 2006.

19. Bowman, A; Azzalini A. Package "sm" [Internet]. 2013. Available from: https://cran.r-project.org/web/packages/sm/sm.pdf

20. MetOffice. Climate Summaries [Internet]. [cited 2017 Nov 22]. Available from: https://www.metoffice.gov.uk/climate/uk/summaries

21. Ofgem. Energy spend as a percentage of total household expenditure (UK) [Internet]. 2017. Available from: https://www.ofgem.gov.uk/data-portal/energy-spend-percentage-total-household-expenditure-uk

22. Hamilton I, Milner J, Chalabi Z, Das P, Jones B, Shrubsole C, et al. Health effects of home energy efficiency interventions in England: a modelling study. BMJ Open [Internet]. 2015 Apr 27;5(4). Available from: http://bmjopen.bmj.com/content/5/4/e007298.abstract

For peer review only

Figure captions

Figure 1. Probability density function of mean winter temperatures in bedroom, living room, and hallway.

Figure 2. Probability density function for proportion of days where 94.4% of days meet the criterion.

Figure 3. Probability density function showing how many hours at night are at least at 18°C.

For peer review only

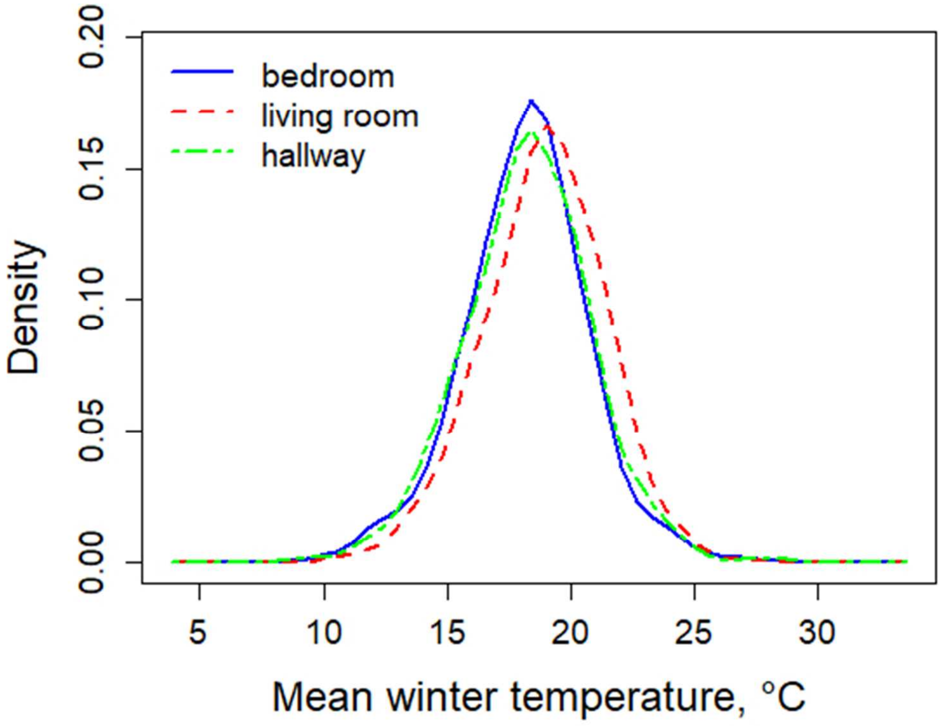

152x130mm (96 x 96 DPI)

only

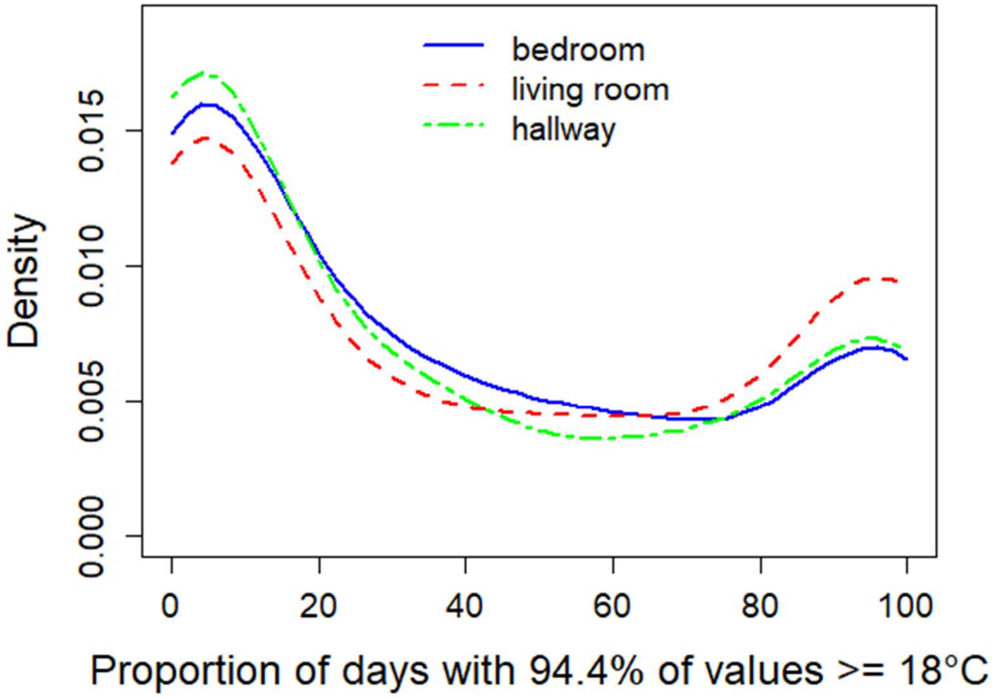

152x130mm (96 x 96 DPI)

only

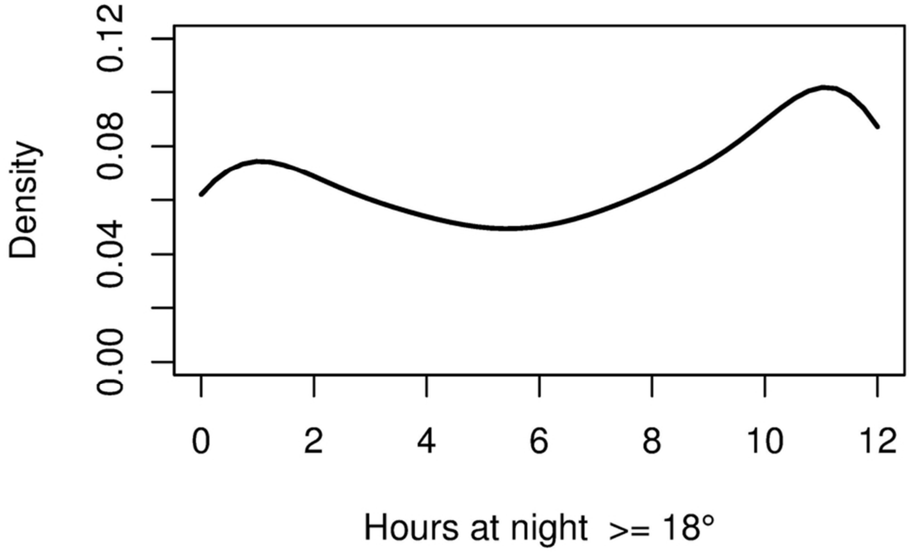

87x65mm (300 x 300 DPI)

STROBE 2007 (v4) Statement—Checklist of items that should be included in reports of *cross-sectional studies*

Section/Topic	Item #	Recommendation	Reported on page #
**Title and abstract**	1	(*a*) Indicate the study's design with a commonly used term in the title or the abstract	1, 4
		(*b*) Provide in the abstract an informative and balanced summary of what was done and what was found	4, 5
**Introduction**			
Background/rationale	2	Explain the scientific background and rationale for the investigation being reported	6
Objectives	3	State specific objectives, including any prespecified hypotheses	7
**Methods**			
Study design	4	Present key elements of study design early in the paper	7
Setting	5	Describe the setting, locations, and relevant dates, including periods of recruitment, exposure, follow-up, and data collection	7
Participants	6	(*a*) Give the eligibility criteria, and the sources and methods of selection of participants	7
Variables	7	Clearly define all outcomes, exposures, predictors, potential confounders, and effect modifiers. Give diagnostic criteria, if applicable	10
Data sources/ measurement	8*	For each variable of interest, give sources of data and details of methods of assessment (measurement). Describe comparability of assessment methods if there is more than one group	9, 10
Bias	9	Describe any efforts to address potential sources of bias	7
Study size	10	Explain how the study size was arrived at	7
Quantitative variables	11	Explain how quantitative variables were handled in the analyses. If applicable, describe which groupings were chosen and why	10
Statistical methods	12	(*a*) Describe all statistical methods, including those used to control for confounding	11,12
		(*b*) Describe any methods used to examine subgroups and interactions	11,12
		(*c*) Explain how missing data were addressed	na
		(*d*) If applicable, describe analytical methods taking account of sampling strategy	na
		(*e*) Describe any sensitivity analyses	na
**Results**			

For peer review only

Participants	13*	(a) Report numbers of individuals at each stage of study—eg numbers potentially eligible, examined for eligibility, confirmed eligible, included in the study, completing follow-up, and analysed	na (did not change)
		(b) Give reasons for non-participation at each stage	na
		(c) Consider use of a flow diagram	na
Descriptive data	14*	(a) Give characteristics of study participants (eg demographic, clinical, social) and information on exposures and potential confounders	8,9
		(b) Indicate number of participants with missing data for each variable of interest	na
Outcome data	15*	Report numbers of outcome events or summary measures	12, 13, 14, 15
Main results	16	(*a*) Give unadjusted estimates and, if applicable, confounder-adjusted estimates and their precision (eg, 95% confidence interval). Make clear which confounders were adjusted for and why they were included	12, 13, 14, 15
		(*b*) Report category boundaries when continuous variables were categorized	9,10
		(*c*) If relevant, consider translating estimates of relative risk into absolute risk for a meaningful time period	na
Other analyses	17	Report other analyses done—eg analyses of subgroups and interactions, and sensitivity analyses	na
**Discussion**			
Key results	18	Summarise key results with reference to study objectives	16,16
Limitations	19	Discuss limitations of the study, taking into account sources of potential bias or imprecision. Discuss both direction and magnitude of any potential bias	15, 16
Interpretation	20	Give a cautious overall interpretation of results considering objectives, limitations, multiplicity of analyses, results from similar studies, and other relevant evidence	16
Generalisability	21	Discuss the generalisability (external validity) of the study results	16
**Other information**			
Funding	22	Give the source of funding and the role of the funders for the present study and, if applicable, for the original study on which the present article is based	17

*Give information separately for cases and controls in case-control studies and, if applicable, for exposed and unexposed groups in cohort and cross-sectional studies.

Note: An Explanation and Elaboration article discusses each checklist item and gives methodological background and published examples of transparent reporting. The STROBE checklist is best used in conjunction with this article (freely available on the Web sites of PLoS Medicine at http://www.plosmedicine.org/, Annals of Internal Medicine at http://www.annals.org/, and Epidemiology at http://www.epidem.com/). Information on the STROBE Initiative is available at www.strobe-statement.org.

For peer review only

BMJ Open

A comparison of indoor temperatures of homes to recommended temperatures and effects of disability and age – an observational, cross-sectional study

Journal:	*BMJ Open*
Manuscript ID	bmjopen-2017-021085.R1
Article Type:	Research
Date Submitted by the Author:	22-Feb-2018
Complete List of Authors:	Huebner, Gesche; UCL, Energy Institute<br>Hamilton, Ian; University College London, UCL Energy Institute<br>Chalabi, Zaid; London School of Hygiene and Tropical Medicine, Social and Environmental Health Research<br>Shipworth, David; Energy Institute<br>Oreszczyn, Tadj; University College London, UCL Energy Institute
**Primary Subject Heading**:	Public health
Secondary Subject Heading:	Health policy
Keywords:	EPIDEMIOLOGY, PUBLIC HEALTH, Adverse events < THERAPEUTICS, Health policy < HEALTH SERVICES ADMINISTRATION & MANAGEMENT

SCHOLARONE™
Manuscripts

For peer review only

Title:

A comparison of indoor temperatures of homes to recommended temperatures and effects of disability and age – an observational, cross-sectional study

Authors:
G. M. Huebner (corresponding author)
Senior Research Associate
g.huebner@ucl.ac.uk
Energy Institute
University College London
London, WC1E 6BT
UK

Ian Hamilton
Reader
Energy Institute
University College London
London, WC1E 6BT
UK

Zaid Chalabi
Associate Professor
Department of Social and Environmental Health Research
London School of Hygiene and Tropical Medicine
London
WC1H 9SH
UK

David Shipworth
Professor
Energy Institute
University College London

For peer review only

London, WC1E 6BT
UK

Tadj Oreszczyn
Professor
Energy Institute
University College London
London, WC1E 6BT
UK

For peer review only

Abstract

Objectives
We examine if temperatures in winter in English homes meet the recommendation of being at least 18°C at all times. We analyse how many days meet this criterion, and calculate the hours per day and night being at/above 18°C. These metrics are compared between households with occupants aged above 64 years or having a long-term disability (LTD), and those younger and without disability

Design.
Cross-sectional, observational.

Setting
England.

Participants.
635 households.

Outcomes measures.
(1) Mean temperatures, (2) proportion of days of the measurement period meeting the criterion, (3) average hours at/above 18°C, (4) average hours at night at/above 18°C.

Results
Mean winter temperatures in the bedroom were M_{BR} = 18.15°C (SD = 2.51), the living room M_{LR} = 18.90°C (SD = 2.46), and the hallway M_{Hall}= 18.25°C (SD = 2.57).

The median number of days meeting the criterion was 19 - 31%. For the living room, more days meet the criterion in the group with a LTD ($M_{disability}$ = 342 vs. $M_{no\_disability}$ = 301; 95% CI: 8 - 74), and with someone over 64 years present ($M_{above64}$ = 341, $M_{below65}$ = 301; 95% CI: 8 - 74).

The median number of hours/day meeting the criterion was 13 - 17. In the living room, households with a disability had more hours at 18°C ($M_{disability}$ = 364, $M_{no\_disability}$ = 297, 95% CI: 17 – 83) as did the older age group ($M_{above64}$ = 347, $M_{below65}$ = 296, 95% CI: 18 – 84). In the hallway, more hours meet the criterion in households with a disability ($M_{disability}$ = 338, $M_{no\_disability}$ = 302, 95% CI: 3 – 70).

247 homes had at least nine hours of at least 18°C at night; no effect of age or disability.

For peer review only

Conclusions.

Many households are at risk of negative health outcomes because of temperatures below recommendations.

Strengths and limitations of the study

- This is the first analysis that allows examining the specific objective of comparing empirical temperature measurements to recommendations.
- The data set used corresponds to a largely representative sample in England.
- Despite outlier correction, it is possible that days were retained in the data set in which the dwelling was empty.
- Only three rooms in the homes were monitored as opposed to every room in a house. Some rooms that were monitored may not have been occupied.
- All three winter months were relatively mild; it is likely that colder winters would mean an even greater discrepancy between recommendations and realized temperatures.

For peer review only

Introduction

The 2016 Cold Weather Plan for England recommended 18°C as day- and night minimum temperature for those 65 and older or anyone with pre-existing medical conditions (1); and a recent systematic review on the link between internal temperatures in homes and health concluded that results from the retrieved studies were sufficient to recommend a temperature of at least 18°C for the whole population at all times (2). The 18°C threshold was judged particularly important for people over 65 years or with pre-existing medical conditions, with a particular emphasis on it being kept at night.

The need for an indoor temperature threshold arises from the burden of excess winter mortality in England; 15% more deaths occur in winter months than non-winter months, corresponding to about 24,000 extra deaths per winter (3), significantly higher than in other European countries (4). Whilst a net of complex factors impacts on winter deaths, the poor state of housing and health inequalities are major reasons for the higher excess winter death rate in England (2,5). Excess winter deaths increase significantly with age of occupants, age of the property, and poorer thermal efficiency ratings, and are associated with lower indoor temperatures (5,6). A meta-analysis on the effects of implementing energy efficiency measures that generally make it easier and more affordable to keep homes warm, showed that there is a small but significant positive effect on health (7).

This paper examines to what extent homes in England meet temperature recommendations in winter by comparing empirical data from 635 homes to the recommendation of 18°C as suggested (2).

Temperatures vary widely between homes and over the course of a day (8). Average temperatures during the heating season in England were 19.3°C for the living room, 18.8°C for the hallway and 18.9°C for the bedroom, based on the Energy Follow-Up survey (9). Whilst these indoor temperatures are above the recommended 18°C, they reflect the average across homes and days. Given the known variability between homes, a substantial number of homes likely had temperatures below the recommendation. Analysis of indoor temperature during cold conditions have shown considerable variability in temperatures among older households that is modified by dwelling energy performance and socio-economic conditions (10).

To our knowledge, no study has assessed to what extent homes in England meet the recommended temperatures. The objectives of this paper are to investigate:

For peer review only

- Number of days in winter meeting the criterion.
- Average number of hours per day meeting the criterion.
- Average number of hours per night meeting the criterion.
- Comparison of the above metrics depending on whether someone in the household has a long-term disability or is over 64 years.

This paper does not aim at explaining reasons behind the differences found, e.g. whether they are due to housing factors, income, personal choice, etc. but focuses on assessing the "status quo" i.e. situation as measured in the study.

Methods

Data.

This study used data from the 2011 Energy Follow Survey (EFUS) commissioned by the Department of Business, Energy and Industrial Strategy (BEIS) (then the Department of Energy and Climate Change) (11), a large-scale cross-sectional national survey in England, and its parent-survey, the English Housing Survey (EHS), a national survey of people's housing circumstances, characteristics and condition. The EFUS survey consisted of an interview survey of a sub-set of households (N = 2,616) that had been first visited as part of the 2010/2011 EHS. A sub-set of those interviewed (N = 943) consented to having temperature loggers in up to three rooms of the house set to record temperatures every 20 minutes from February 2011 to January 2012. It is not known if there was any systematic difference in temperature between those who consented to loggers and those who did not, this is unlikely given that the households with loggers were broadly representative in regards to Census data (see Table 1).

The linked data sets were explicitly made available by BEIS for this research project. Parts of the dataset used in this study remain private (i.e. the high-resolution temperature data and the connection identifier between the EFUS and EHS). The non-linked data sets and summarised temperature data are accessible via the UK Data Archive. As this paper constitutes secondary data analysis, no ethical approval was required and no personal data (i.e. identifying individuals) was available or used.

Valid temperature data was obtained from N = 823 households (see (11) for details). For this paper, only those N = 760 households with three rooms temperature monitored (bedroom, living room, hallway) were included. 105 households were excluded because of changes to the household or home since the last EHS. Hence, the final sample size on which all analyses

For peer review only

are based is N = 635 homes with approximately national representativeness on geographical location, tenure and dwelling type (see Table 1).

Table 1. Comparison of sample characteristics to 2011 Census data.

	N in sample	% in sample	% in 2011 census
**Region** (12)			
North East	44	6.93	4.90
North West	103	16.22	13.30
Yorkshire & Humber	83	13.07	9.97
East Midlands	53	8.35	8.55
West Midlands	58	9.13	10.57
East	88	13.86	11.03
London	46	7.24	15.42
South East	101	15.91	16.29
South West	59	9.29	9.98
**Dwelling type** (13)			
Detached	153	24.09	22.30
Semi-detached	204	32.13	30.70
Terraced (including end-terrace)	178	28.03	24.50
Purpose-built flats	86	13.54	16.70
Converted flat	14	2.20	4.30
In commercial building	0	0.00	1.10
Caravan, mobile home etc.	0	0.00	0.40
**Tenure** (14)			
Owned outright	192	30.24	30.60
Owned with a mortgage / loan	211	33.23	32.80
Shared ownership	na	na	0.80
Rented from council (Local Authority)	82	12.91	9.40
Social rented: Other	94	14.80	8.30
Private rented	56	8.82	16.80
Living rent free	na	na	1.30

For peer review only

Tenure is the only variable showing some larger discrepancy between the sample and census, with 'social rented: other' over-represented by about 7% and 'privately rented' under-represented by about 8%. Given that socially rented accommodation is generally the best in terms of energy efficiency, and privately rented accommodation the worst, this mismatch might indicate that in a truly representative sample the criterion of at least 18°C would be met to a slightly lesser extent.

Survey interview data for EHS and EFUS.

Data were collected through computer-assisted personal interviewing in the home of the respondent. For the purpose of this study, only questions relating to age of the householder and their self-reported health were analysed.

Respondents were asked if they and other household members, where applicable, had any long-standing physical or mental health condition. If the question condition was affirmed, the interviewer asked for a specification (Table 2).

Table 2. Prevalence of long-term disabilities (LTD) in the sample.

Long-term disability type	Number of households in which prevalent
Vision	28
Hearing	24
Learning	12
Heart	75
Breathing	92
Mobility	146
Mental	35
Other	165
Don't know	4

Note that in some households multiple LTD existed, i.e. the 581 occurrences listed here were distributed across 369 households.

Of the N = 635 households, N = 369 reported one or more long-term disability. For the purpose of this study, only the dichotomized variable of "any long-term disability" vs "no

For peer review only - http://bmjopen.bmj.com/site/about/guidelines.xhtml

long-term disability" was used, irrespective of the type of condition and total number of individuals with LTDs in one household. Any LTD indicates vulnerability in the household and an adaptation of the environment would be required.

The second variable of interest was age of the oldest household member; age was dichotomized into "64 years and younger" and "65 years and older", because 65 years was the cut-off used for the specific recommendations on indoor temperatures (1). Among the N = 635 households, N = 206 dwellings had the oldest household member of age 65 or older.

Temperature recordings.

Temperatures were recorded every 20 minutes using modified TinyTag Transit 2 data loggers, that have an accuracy of +/-0.2°C, and a resolution of 0.01°C (11). The temperature loggers were usually installed by the interviewer at the end of the EFUS interview, on an internal wall, away from heat sources and direct sunlight, at a height accessible by the householder but out of reach of small children (11).

Temperature recordings for February 2011, December 2011, and January 2012 were used, i.e. those months considered as winter by the Office for National Statistics (15) for which temperature data were available (16). Note, the specific months monitored were mild compared to historic years, with February 2011 being 1.7°C milder across the UK than the UK average 1981-2010, and December 2011 and January 2012 being both 1°C milder than the 1981-2010 average (17). Internal temperatures are dependent on external temperatures, hence the temperatures during colder years will be significantly lower than presented here. For every dwelling, an extreme value correction was performed on the combined temperature data from the three months where any data point more than 1.5 interquartile ranges (IQRs) below the first quartile was removed as extremely low temperatures might reflect absence from the home. The median numbers of extreme values removed were 13, 2, and 8, for bedroom, living room, and hallway, respectively.

Derived variables.

Four outcome variables were constructed from the recorded temperature readings.

a) Mean temperature for each room over the winter period.

For each dwelling and room, the average temperature across the three winter months was calculated.

b) Days with temperatures at or above 18°C

We calculated the number of days in which homes met the criterion of being at least 18°C continuously. Whilst a strict interpretation of the recommendation would mean that 100% of all measurements need to be at 18°C or above (i.e. all 72 measurements), we relaxed the assumption to 94.4% of all measurements (i.e. 68 out of 72 measurement points). This is meant to take into account that brief drops in temperature are entirely plausible, e.g. due to window or door opening.

For each home, on each day, and in each room, we checked at each measurement point if the temperature was at least 18°C, with a 1 recorded if it was and a 0 if it was not. The values for each day were summed up and divided by the total number of measurements per day. If 68 measurements were at 18°C or above, then the resulting value would be 68/72 = 0.94, i.e. 94.4%. We calculated the percentage of days for which the temperature measurements during the day had 94.4% of values at 18°C or above.

The percentage of days meeting the criterion is reported instead of the absolute number as some homes did not have temperature data recordings for all 90 days (median was 86 days).

c) Hours at or above 18°C

For each home, on each day, and in each room, we calculated the average number of hours for which the temperature was at least 18°C per a 24-hour period. We checked if consecutive measurement, i.e. 20-minute segments, were both at least 18°C, where each day lasted from midnight to midnight the next day. This meant that two days (30th of January, 2012, 28th of February, 2011) were excluded from analysis as there was no subsequent day. For each home, we averaged the estimated daily temperature metrics across all days, separately for each room.

d) Hours at or above 18°C during night

We defined night-time as lasting from 8 pm to 8 am next day to take into account that people sleep at different times, and identified whether 20-minute segments of temperature readings (i.e. two consecutive measurement) were at 18°C or above within the 12-hour time window. As above, two days were excluded. For each home, we averaged the estimated nightly temperature metrics across all days. Only the bedroom was considered.

Hence, four outcome variables were derived from the raw data for each dwelling. The first three, average temperatures (a), proportion of days meeting the criterion (b), and hours

For peer review only

meeting the criterion (c), were calculated separately for each room. The final outcome variable, hours meeting the criterion at night, was only calculated for the bedroom, assuming that that is where people slept.

Statistical analysis.

For the normally distributed variable ‘mean temperature’ (outcome variable a), a repeated measures ANOVA was used to test for differences between rooms, and a generalized linear model (GLM) with the fixed factors age and disability status and their interaction to test if temperatures differed depending on those variables. Post-hoc comparisons were Bonferroni adjusted.

The non-normally distributed outcome variables (b) – (d) were analysed using ANOVA on ranks (18) whereby data are transformed into ranks (averaged in the case of ties) over the entire data set, and then a parametric ANOVA is applied to the ranks. The rank 1 was assigned to the lowest value, i.e. to zero days meeting the criterion; a higher mean rank value indicates more days meeting the criterion. The main effects of age and disability were tested and their interaction. The presence of an interaction effect is to be interpreted with greatest caution as the procedure is associated with an increase in Type 1- error (i.e., claiming statistical significance where there is none, see e.g. (19)); however, if no interaction effect is found, it can be assumed that indeed, there isn’t one.

Additionally, for days at or above 18°C (outcome variable (b)), relative risk was calculated following (20) for the rooms where disability or age had a significant effect to be able to easily articulate how much more likely those more vulnerable were to live at the criterion.

Patient and Public Involvement

As this paper constitutes secondary data analysis, there was no involvement of patients or the public.

Results

Mean temperature for each room over the winter period.

Across all dwellings, mean temperatures in the bedroom were M_{BR} = 18.15°C (SD = 2.51), the living room M_{LR} = 18.90°C (SD = 2.46), and the hallway M_{Hall}= 18.25°C (SD = 2.57). A repeated measures ANOVA showed a main effect of room type, $F(2, 1268) = 58.41$, $p < .001$. Post-hoc comparisons showed the living room was significantly warmer than the bedroom (p

For peer review only

< .001; mean difference: .75, 95% CI for difference: .94 – .57) and hallway ($p < .001$; mean difference: .65; 95% CI for difference: .57 –. 94) which did not differ significantly from each other.

Figure 1 shows the probability density function (PDF) of the mean temperatures for the three rooms, created using the R package 'sm' (21). The PDF is best understood through the area underneath it. The area underneath the PDF of a continuous random variable between two values gives the probability that the random variable is between those values. The total area underneath the PDF over the whole range of values of the random variable is unity.

<< insert Figure 1 about here >>

Figure 1 indicates a wide spread in mean temperatures. Whilst the average temperature (across days and homes) in all three rooms is slightly above 18°C, in a substantial number of homes it was below 18 °C. In the case of the bedroom, 286 dwellings (45%) had an average temperature below 18 °C, in the living room 209 (33%), and in the hallway 278 dwellings (44%).

In the bedroom, only the effect of disability was significant [$F(1, 631) = 4.38$, $p = .037$] with higher temperatures in the group with disability ($M_{disability}$ = 18.35°C; $M_{no\_disability}$ = 17.87°C; 95% CI for difference: .03 – .94). For the hallway, again only the effect of disability was significant [$F(1, 631) = 7.64$, $p = .006$] with higher temperatures in the disability group ($M_{disability}$ = 18.58°C, $M_{no\_disability}$ = 17.93°C). There was a strong trend for higher temperatures in the homes of the older age group ($p = .059$). In the living room, both the main effects of age [F(1, 631 = 12.39, $p < .001$] and disability [$F(1, 631) = 15.53$, $p <.001$] were statistically significant. Temperature were higher in the group with disability ($M_{disability}$ = 19.37°C; $M_{no\_disability}$ = 18.50°C; 95% CI for difference: .44 – 1.30) and in the older age group ($M_{above64}$ = 19.32°C; $M_{below65}$ = 18.55°C; 95% CI for difference: .34 – 1.21).

Days with temperatures above 18°C

We analysed the number of days during the winter on which dwellings met the indoor temperature criterion. Figure 2 shows the probability density function of the distribution for the three rooms.

<< insert Figure 2 about here >>

For peer review only

Figure 2 indicates that the largest share of homes do not meet the criterion but that a substantial number of homes meet it on 90-100% of days. For the bedroom, 11% of homes meet the criterion on all days, and 17% on more than 90% of days. For the living room, the numbers are 15% and 24%, respectively, and for the hallway 12% and 17%. The median number of days that indoor temperatures meet the criterion on all days is Md_{BR} = 22.6% of days, MD_{LR} = 31.1%, and Md_{Hall} = 18.9%.

The ANOVA for ranks in the bedroom showed neither a main effect of age or disability nor an interaction effect. For the living room both the main effects of disability [$F(1, 631) = 6.00$, $p = .015$] and age [$F(1, 631) = 6.06$, $p = .0114$] were significant, with a higher share of days meeting the criterion in the group with a LTD ($M_{disability}$ = 342 vs. $M_{no\_disability}$ = 301; 95% CI for difference: 8 - 74) and with someone over 64 years present ($M_{above64}$ = 341, $M_{below65}$ = 301; 95% CI for difference: 8 - 74). Of those households with LTD, 26.8% had a continuous temperature above 18°C on 90% of days or more compared to 20.7% for those without LTD. Expressed as a relative risk (20), people with LTD are 1.30 times more likely to be living in dwellings where the temperature is consistently over 18°C compared to those without LTD, and people who are 65 years and above are 1.56 more likely than those below 65 years.

For the hallway, there were no significant effects. However, there was suggestive evidence of a trend towards more days meeting the criterion in the group with a long-term disability (p =.064).

Number of hours at which temperatures are at or above 18°C

The number of hours at or above 18°C were non-normally distributed with peaks at either extreme of zero and 24 hours. The median number of hours at / above 18°C was Md_{BR} = 14:01 hours per day, Md_{LR} = 16:57 hours, and Md_{Hall} = 13:24 hours. Table 3 shows for how many hours in each room, depending on disability and age group, the criterion was met.

Table 3. Median number of hours with temperatures at the criterion for the three rooms separated by disability and age group.

	Disability status		Age	
	No LT disability	LT disability	Below 65 years	Above 64 years

For peer review only

Bedroom	13:10 hrs	14:05 hrs	12:56 hrs	15:09 hrs
Living room	15:13 hrs	17:59 hrs	15:37 hrs	20:01 hrs
Hallway	10:58 hrs	14:52 hrs	12:35 hrs	16:01 hrs

In the bedroom, there were no significant main or interaction effects. In the living room, both the main effect of disability [$F(1, 631) = 8.89$, $p = .003$] and of age [$F(1, 631) = 9.28$, $p = .002$] were significant, with more hours at or above 18°C in those households occupied by individuals with a disability ($M_{disability} = 364$, $M_{no\_disability} = 297$, 95% CI for difference: 17 – 83) and in the older age group ($M_{above\ 64} = 347$, $M_{below65} = 296$, 95% CI for difference: 18 – 84). In the hallway, the main effect of disability was significant [$F(1, 631) = 4.53$, $p = .034$] and the effect of age approached significance ($p = .073$) with again more hours meeting the criterion in the group with a LTD ($M_{disability} = 338$, $M_{no\_disability} = 302$, 95% CI for difference: 3 – 70) and the older group.

Night temperatures

We subsequently tested if the recommendation of nine hours of 18°C at night time was met (see Figure 3).

<< insert Figure 3 about here >>

Across the full sample, 247 homes (38.9%) had at least nine hours of temperatures of at least 18°C at night and 101 (15.9%) homes had less than one hour at the criterion. The median was 7:08 hrs.

An ANOVA on ranks showed neither a main effect of disability nor of age, and no interaction.

Discussion

This study is the first to establish whether measured temperature data in homes corresponds to the recommended temperatures, in the general population and the subgroups of those with a LTD and / or aged 65 and above. Whilst average temperatures across homes in this sample

For peer review only

is slightly above 18°C, the wide variability means that many homes have lower temperatures. Depending on room type, the recommended indoor temperature of 18°C was only met on 19 – 31% of days during the three studied mild winter months. Only 5% to 9% of homes met the criterion of being at least 18°C throughout the day, with up to 22% of homes meeting the criterion if set as having the recommended temperature throughout the day for at least 90% of days. Those with a disability and old age were 1.3 and 1.56 more likely to meet the condition than those without disability and the younger age group. The median number of hours per day at or above 18°C was 17 for the living room, and 14 and 13 ½, respectively, for bedroom and hallway. The median number of hours meeting the criterion were one to three hours higher in households with a disability or aged 65 and above. At night, 37% of homes had temperatures of at least nine hours at 18°C or more, with the median being 7 hrs, with no effect of age group or disability.

In summary, the majority of measures employed showed that the recommendation was not met, neither in the overall sample, nor within the subsamples of those more vulnerable to effects of cold.

Limitations and strengths of this study

Despite outlier correction, it is possible that days were retained in the data set in which the dwelling was empty, leading to an underestimation of the criterion being met assuming that it only holds for occupied times. Only three rooms in the homes were monitored as opposed to every room in a house. The study is cross-sectional, and cannot add evidence on whether low temperatures are associated with poor health outcomes. All three winter months were relatively mild (mean temperatures in February 2011 1.7 °C above the 1981-2010 average; in December 2011 1.0 °C above the 1981-2010 average; January 2012 1.0 °C above the 1981-2010 average (17)); it is likely that colder winters would mean even lower prevalence of 18°C. Households consented to having temperature loggers installed; it is possible that temperatures in those households were either higher or lower than in those not giving consent.

This paper is the first analysis that allows examining the specific objective of comparing empirical temperature measurements to recommendations, showing a significant discrepancy and the need for action. The data set used corresponds to a largely representative sample in England; hence, results likely are generalizable to the whole of England.

For peer review only

Conclusions

In summary, data showed that the majority of homes do not meet the recommendation, neither across the whole sample or within the vulnerable subgroups. If living in homes below the temperature threshold is a determinant of cold-related ill health then many English households are at risk of developing negative health outcomes. If this exposure presents a high risk to health, then substantial action is needed to increase temperatures in homes, be it through improvements in building fabric, extended use of heating systems, or increased thermostat set points.

From an energy demand perspective, energy use in buildings would increase substantially when keeping all homes at 18°C continuously. Without improvement in the energy performance of buildings, e.g. through fabric insulation and greater efficiency of heating systems, this outcome would result in an increase in heating energy use and move away from the UK's energy efficiency goals. Hence, implementing new and stricter policies on retrofitting are needed. The UK has been dubbed 'the cold man of Europe' given that in comparison to other European countries, it has one of the highest level of fuel poverty and some of the most inefficient housing stock, with 21 of out of 26 million dwellings rated as 'D' or below on their energy performance certificate (22). Energy efficiency interventions, have been shown to increase daytime living room temperatures by 1.6 °C, and night time bedroom temperatures by 2.8 °C (23). Increased energy efficiency can bring the risk of higher temperatures in summer which might also be detrimental for health (7).

There is also the question of whether individuals can afford to increase fuel expenditure to achieve the stated indoor temperature threshold. Mean energy expenditure was 4.4% of total household expenditure, with a substantially higher proportion of 9.7% in the lowest income decile (24). Spending on fuel to increase temperatures would result in a greater proportion of household resources allocated to fuels. This increase in spending could result in a net cost-benefit if the health impacts were accounted for in these calculations (25)– but householders might not realize this directly.

Contributor ship statement

GMH and IH conceived the study.

GMH conducted the analysis.

ZC advised on the analysis.

GMH wrote the paper.

For peer review only

IH, ZC, TO, and DS proofread the manuscript multiple times.

Competing interests

The authors declare no competing interests.

Funding

Funding for this research was provided by the Research Councils UK (RCUK) Centre for Energy Epidemiology, grant reference EP/K011839/1.The funders were not involved in any aspects of the research.

Data sharing agreement

The linked data sets were explicitly made available by BEIS for this research project. Parts of the dataset used in this study remain private (i.e. the high resolution temperature data and the connection identifier between the EFUS and EHS to link dwelling and household features). The individual, non-linked data sets and the summarised temperature data are available on UK Data Archives.

For peer review only

References

1. Public Health England. The Cold Weather Plan for England Protecting health and reducing harm from cold weather About Public Health England. 2015;(October).

2. Jevons R, Carmichael C, Crossley A, Bone A. Minimum indoor temperature threshold recommendations for English homes in winter – A systematic review. Vol. 136, Public Health. 2016. p. 4–12.

3. Office for National Statistics. Excess Winter Mortality in England. 2014;14:22.

4. Fowler T, Southgate RJ, Waite T, Harrell R, Kovats S, Bone A, et al. Excess winter deaths in Europe: A multi-country descriptive analysis. Eur J Public Health. 2015;25(2):339–45.

5. Wilkinson P, Landon M, Armstrong B, Stevenson S, Pattenden S, Mckee M, et al. Cold comfort: The social and environmental determinants of excess winter deaths in England , 1986-96. 2001;34.

6. NICE. Excess cess winter deaths and illness and the health risks associated with cold homes. 2015.

7. Maidment CD, Jones CR, Webb TL, Hathway EA, Gilbertson JM. The impact of household energy efficiency measures on health: A meta-analysis. Energy Policy [Internet]. 2014 Feb 1 [cited 2018 Jan 31];65:583–93. Available from: https://www.sciencedirect.com/science/article/pii/S030142151301077X

8. Huebner GM, McMichael M, Shipworth D, Shipworth M, Durand-Daubin M, Summerfield A. The reality of English living rooms - A comparison of internal temperatures against common model assumptions. Energy Build [Internet]. 2013;66:688–96. Available from: http://dx.doi.org/10.1016/j.enbuild.2013.07.025

9. Hulme J, Beaumont A, Summers C. Energy Follow-Up Survey 2011, Report 1: Summary of findings. BRE behalf DECC. 2013;(289605).

10. Hamilton IG, O'Sullivan A, Huebner G, Oreszczyn T, Shipworth D, Summerfield A, et al. Old and cold? Findings on the determinants of indoor temperatures in English dwellings during cold conditions. Energy Build. 2017 Apr;141:142–57.

11. BRE. Energy Follow-Up Survey 2011, Report 11 : Methodology. 2013;(288851):1–

For peer review only

120.

12. ONS. KS101EW Usual resident population, local authorities in England and Wales.

13. ONS. KS401EW Dwellings, household spaces and accommodation type, local authorities in England and Wales (Excel sheet 305Kb).

14. ONS. KS402EW Tenure, local authorities in England and Wales (Excel sheet 270Kb).

15. MetOffice. Winter 2011/12.

16. BRE. Report 2 : Mean household temperatures. 2013.

17. MetOffice. Climate Summaries.

18. Conover WJ, Iman RL. Rank Transformations as a Bridge between Parametric and Nonparametric Statistics Rank Transformations as a Bridge Between Parametric and Nonparametric Statistics. Am Stat. 1981;

19. Higgins JJ, Tashtoush S. An aligned rank transform test for interaction. Nonlinear World. 1994;

20. Altmann DG. Practical Statistics for Medical Research. Chapman & Hall; 2006.

21. Bowman, A; Azzalini A. Package "sm." 2013.

22. Association for the Conservation of Energy. COLD MAN OF EUROPE 2015 UPDATE The Cold Man of Europe – 2015 [Internet]. 2015 [cited 2018 Feb 1]. Available from: http://www.ukace.org/wp-content/uploads/2015/10/ACE-and-EBR-briefing-2015-10-Cold-man-of-Europe-update.pdf

23. Oreszczyn T, Hong SH, Ridley I, Wilkinson P. Determinants of winter indoor temperatures in low income households in England. Energy Build [Internet]. 2006 Mar 1 [cited 2018 Feb 1];38(3):245–52. Available from: https://www.sciencedirect.com/science/article/pii/S0378778805000964

24. Ofgem. Energy spend as a percentage of total household expenditure (UK). 2017.

25. Hamilton I, Milner J, Chalabi Z, Das P, Jones B, Shrubsole C, et al. Health effects of home energy efficiency interventions in England: a modelling study. BMJ Open. 2015 Apr;5(4).

For peer review only

Figure captions

Figure 1. Probability density function of mean winter temperatures in bedroom, living room, and hallway.

Figure 2. Probability density function for proportion of days where 94.4% of days meet the criterion.

Figure 3. Probability density function showing how many hours at night are at least at 18°C.

For peer review only

For peer review only

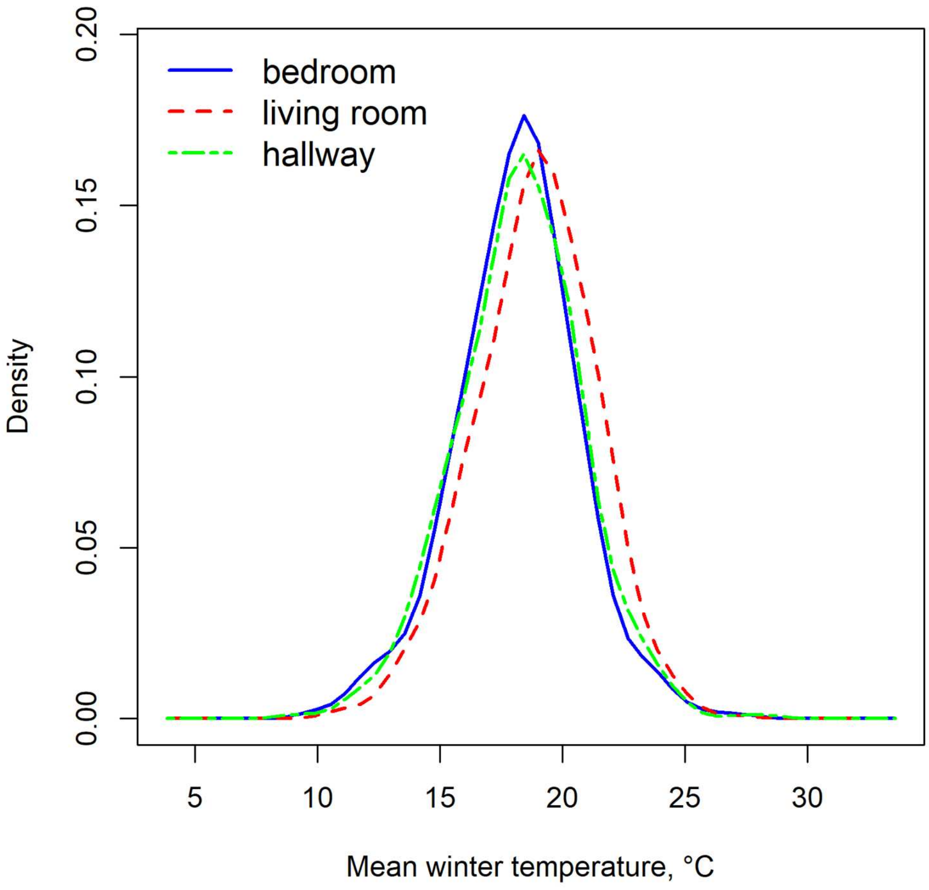

Figure 1. Probability density function of mean winter temperatures in bedroom, living room, and hallway.

147x152mm (300 x 300 DPI)

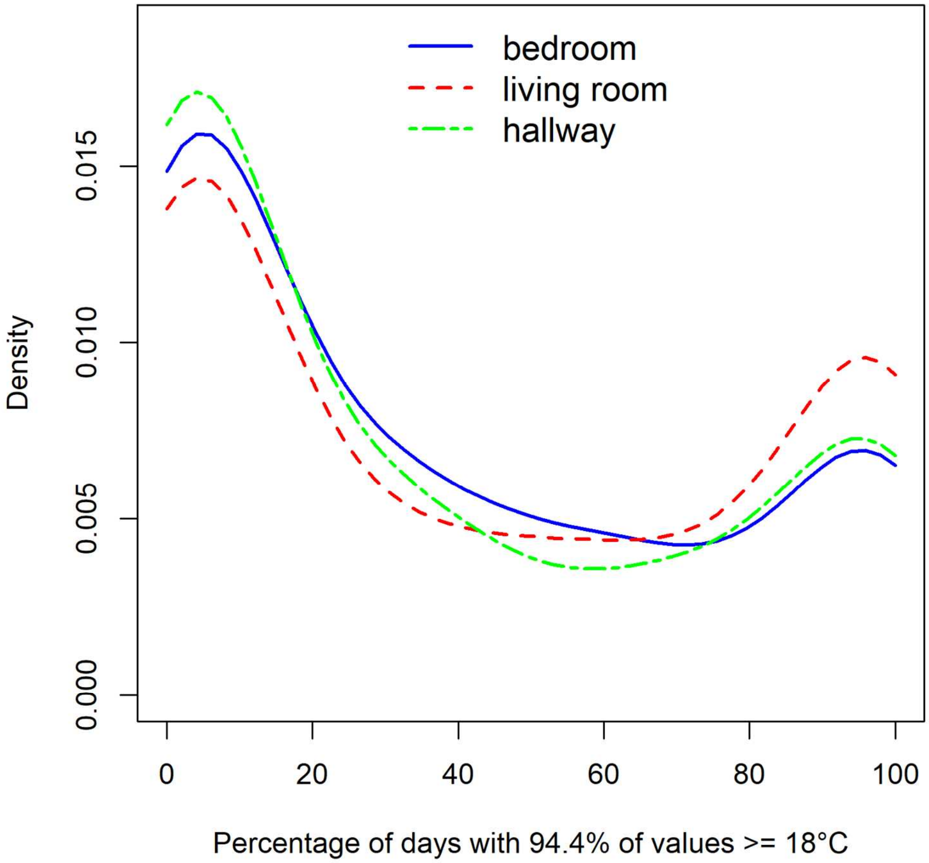

Figure 2. Probability density function for percentage of days where 94.4% of days meet the criterion.

147x152mm (300 x 300 DPI)

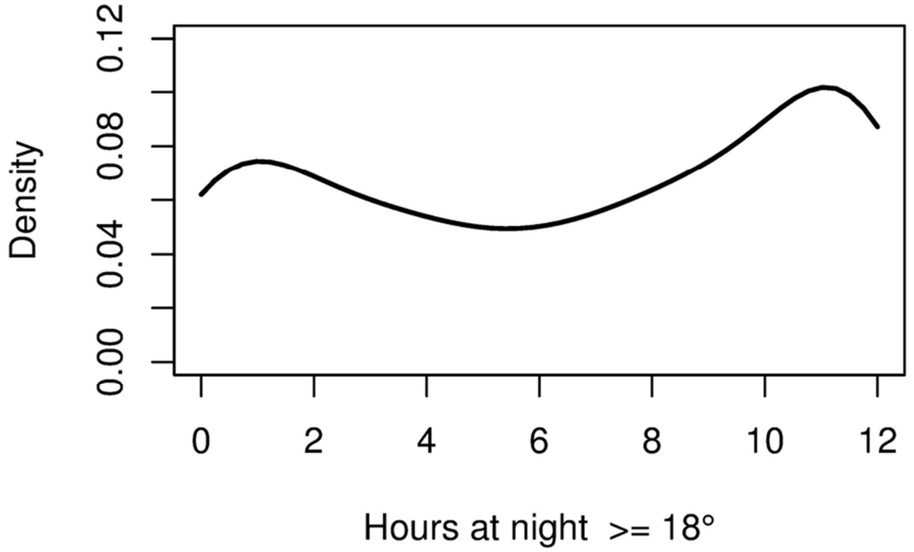

87x65mm (300 x 300 DPI)

For peer review only - http://bmjopen.bmj.com/site/about/guidelines.xhtml

STROBE 2007 (v4) Statement—Checklist of items that should be included in reports of *cross-sectional studies*

Section/Topic	Item #	Recommendation	Reported on page #
**Title and abstract**	1	(*a*) Indicate the study's design with a commonly used term in the title or the abstract	1, 4
		(*b*) Provide in the abstract an informative and balanced summary of what was done and what was found	4, 5
**Introduction**			
Background/rationale	2	Explain the scientific background and rationale for the investigation being reported	6
Objectives	3	State specific objectives, including any prespecified hypotheses	7
**Methods**			
Study design	4	Present key elements of study design early in the paper	7
Setting	5	Describe the setting, locations, and relevant dates, including periods of recruitment, exposure, follow-up, and data collection	7
Participants	6	(*a*) Give the eligibility criteria, and the sources and methods of selection of participants	7
Variables	7	Clearly define all outcomes, exposures, predictors, potential confounders, and effect modifiers. Give diagnostic criteria, if applicable	10
Data sources/ measurement	8*	For each variable of interest, give sources of data and details of methods of assessment (measurement). Describe comparability of assessment methods if there is more than one group	9, 10
Bias	9	Describe any efforts to address potential sources of bias	7
Study size	10	Explain how the study size was arrived at	7
Quantitative variables	11	Explain how quantitative variables were handled in the analyses. If applicable, describe which groupings were chosen and why	10
Statistical methods	12	(*a*) Describe all statistical methods, including those used to control for confounding	11,12
		(*b*) Describe any methods used to examine subgroups and interactions	11,12
		(*c*) Explain how missing data were addressed	na
		(*d*) If applicable, describe analytical methods taking account of sampling strategy	na
		(*e*) Describe any sensitivity analyses	na
**Results**			

For peer review only

Participants	13*	(a) Report numbers of individuals at each stage of study—eg numbers potentially eligible, examined for eligibility, confirmed eligible, included in the study, completing follow-up, and analysed	na (did not change)
		(b) Give reasons for non-participation at each stage	na
		(c) Consider use of a flow diagram	na
Descriptive data	14*	(a) Give characteristics of study participants (eg demographic, clinical, social) and information on exposures and potential confounders	8,9
		(b) Indicate number of participants with missing data for each variable of interest	na
Outcome data	15*	Report numbers of outcome events or summary measures	12, 13, 14, 15
Main results	16	(*a*) Give unadjusted estimates and, if applicable, confounder-adjusted estimates and their precision (eg, 95% confidence interval). Make clear which confounders were adjusted for and why they were included	12, 13, 14, 15
		(*b*) Report category boundaries when continuous variables were categorized	9,10
		(*c*) If relevant, consider translating estimates of relative risk into absolute risk for a meaningful time period	na
Other analyses	17	Report other analyses done—eg analyses of subgroups and interactions, and sensitivity analyses	na
**Discussion**			
Key results	18	Summarise key results with reference to study objectives	16,16
Limitations	19	Discuss limitations of the study, taking into account sources of potential bias or imprecision. Discuss both direction and magnitude of any potential bias	15, 16
Interpretation	20	Give a cautious overall interpretation of results considering objectives, limitations, multiplicity of analyses, results from similar studies, and other relevant evidence	16
Generalisability	21	Discuss the generalisability (external validity) of the study results	16
**Other information**			
Funding	22	Give the source of funding and the role of the funders for the present study and, if applicable, for the original study on which the present article is based	17

*Give information separately for cases and controls in case-control studies and, if applicable, for exposed and unexposed groups in cohort and cross-sectional studies.

Note: An Explanation and Elaboration article discusses each checklist item and gives methodological background and published examples of transparent reporting. The STROBE checklist is best used in conjunction with this article (freely available on the Web sites of PLoS Medicine at http://www.plosmedicine.org/, Annals of Internal Medicine at http://www.annals.org/, and Epidemiology at http://www.epidem.com/). Information on the STROBE Initiative is available at www.strobe-statement.org.

For peer review only
